# Arm-hand BOOST (AHA-BOOST) therapy to improve recovery of the upper limb after stroke: rationale and description by means of the TIDieR checklist

**DOI:** 10.3389/fneur.2025.1599762

**Published:** 2025-09-10

**Authors:** Marc Michielsen, Liesel Cornelis, Lisa Cruycke, Ann De Smedt, Maaike Fobelets, Koen Putman, Maaiken Vander Plaetse, Geert Verheyden, Sarah Meyer

**Affiliations:** ^1^Jessa Hospital, Rehabilitation Campus REVA HERK, Herk-de-Stad, Belgium; ^2^Department of Rehabilitation Sciences, KU Leuven, Leuven, Belgium; ^3^I-CHER, Department of Public Health, Vrije Universiteit Brussel, Brussel, Belgium; ^4^Research Centre for Digital Medicine, Vrije Universiteit Brussel, Brussel, Belgium; ^5^STIMULUS Research Group, Vrije Universiteit Brussel, Brussel, Belgium; ^6^Cluster Neurosciences, Center for Neurosciences (C4N), Vrije Universiteit Brussel, Brussels, Belgium; ^7^Department of Physical Medicine and Rehabilitation, Universitair Ziekenhuis Brussel, Jette, Belgium; ^8^Department of Teacher Education, Vrije Universiteit Brussel, Brussels, Belgium; ^9^Department of Public Health, Biostatistics and Medical Informatics Research Group, Faculty of Medicine and Pharmacy, Vrije Universiteit Brussel, Brussels, Belgium; ^10^Leuven Brain Institute, KU Leuven, Leuven, Belgium

**Keywords:** stroke, upper extremity, therapy program, recovery, rationale, TIDieR

## Abstract

**Purpose:**

This methods article provides a detailed description of the Jessa AHA-BOOST program; an intensive, comprehensive, specific arm-hand therapy program for patients after stroke.

**Materials and methods:**

The TIDieR (Template for intervention description and replication) checklist was used for the overview, which includes 7 topics: ‘Why’, ‘What’, ‘Who provided’, ‘How’, ‘Where’, ‘When and How much’ and ‘Tailoring’. Particularly the rationale for the program is extensively described.

**Results:**

The AHA-BOOST therapy program is developed for patients with mild to moderate impairments in the upper limb after stroke. It offers a 4-week tailored treatment program consisting of daily 1-h group sessions and weekly individual robot-assisted therapy. The sessions are built on neurophysiological and kinematic knowledge of reaching and grasping and on the principles of motor learning. The most important aspects in the content of the AHA-BOOST program are: (1) Neurophysiology; (2) Sequences of reaching and grasping; (3) De-weighting; and (4) Hand orientation.

**Conclusion:**

In a phase II RCT, the AHA-BOOST program proved to be feasible and safe and suggests positive, clinical meaningful effects on arm and hand function. A phase III RCT including clinical, health economic and process evaluations of the AHA-BOOST program is currently ongoing.

## 1 Introduction

Reduced arm function causes a decrease of quality of life in people with stroke (1). Initially, 80% of people with stroke have an impairment of arm-hand function. These impairments persist in 55% of chronic people with stroke (2). Only 5–20% demonstrate complete recovery (3). It is therefore important to provide adequate upper limb rehabilitation, starting early post stroke, with continued attention in the sub-acute and chronic phase.

In 2015, the Jessa Sint-Ursula (JSU)—diagram (4) was developed with the aim of structuring the approach for the treatment of the upper limb in acquired brain injuries. It is emphasized that an early start of rehabilitation of the upper limb is crucial in relation to the functional outcome of reaching and grasping. Additionally, the diagram draws attention to stratification of patients according to their functional potential, which is needed to give patient-tailored programs. In the diagram, three patient groups are defined based upon the degree of recovery and relearning potential of the arm and hand: a basic group (severe impairment), the arm-hand boost (AHA-BOOST) group (moderate to mild impairment) and a functional group (very mild impairment). An intensive, comprehensive, specific arm-hand therapy was developed for the AHA-BOOST group by the rehabilitation team of Jessa hospital: the Jessa AHA-BOOST program. This program builds upon neurophysiological and kinematic knowledge of reaching and grasping and upon the principles of motor learning.

In this methods article, we present a detailed description of the program, with a focus on the theoretical background of the content, using a comprehensive elaboration of TIDieR (Template for intervention description and replication) (5) for the Jessa AHA-BOOST program.

## 2 Materials and equipment

### 2.1 Procedures

AHA-BOOST group sessions are focused around the four key aspects (extensively described below): neurophysiology, sequences of reaching and grasping, de-weighting of the arm and orientation of the hand towards objects. Each of the sessions is tailored to the individual patient, based upon the ongoing assessment of the therapists, position within the JSU diagram (4), discussion within the group of therapists, standardized assessment [Model of Bobath Clinical Practice (MBCP)] (6) and individual treatment goals of the patient.

Group sessions are conducted with a maximum of four patients, supervised by two trained therapists (physiotherapist and occupational therapist). Sessions may begin with a brief mobilization to prepare for the exercises, followed by approximately eight exercises. These exercises are designed for the patient to perform with minimal support of a therapists. By adapting the environment, the four key aspects are integrated. Additionally, patients perform 1 h per week individual robot-assisted therapy. During the full session the amount of assistance provided by the devices is based on the patients’ active possibilities.

### 2.2 Materials

The material used in the AHA-BOOST group sessions include standard rehabilitation material such as different sizes of balls, cones, hoops, beads, and daily objects. For the individual robot-assisted therapy, several upper limb rehabilitation technologies can be used, such as the Armeo Power® (Hocoma, Switzeland), Myro® (Tyromotion, Austria), Amadeo® (Tyromotion, Austria), or similar, depending upon therapy goals, abilities of patients and availability of the systems. The therapy goals and abilities of the patients are determined together with the patient by a multidisciplinary team and are based upon recommended clinical outcome measures, such as Fugl-Meyer assessment for upper extremity (FMA-UE), action research arm test, stroke upper limb capacity scale or goal attainment scale, or similar, aligned with consensus-based core recommendations from the Stroke Recovery and Rehabilitation Roundtable (7). In the AHA-BOOST therapy program, we primarily focus on performance-based measures to obtain detailed, standardized, and objective assessments of motor function and movement quality under controlled conditions. Our aim is to evaluate potential therapy effects within the structured context of the inpatient rehabilitation setting. According to the ICF model, we include both impairment and activity (capacity) outcome measures, based on international consensus. We are aware that real-world upper limb use is a key outcome for persons with stroke and acknowledge that within-laboratory capacity measures alone may not fully capture spontaneous functional use or performance in daily life. Future trials should therefore consider to include a meaningful measure of real-world upper limb use such as for instance the Motor Activity Log (MAL) or wearable sensor technology.

## 3 Methods

The Jessa AHA-BOOST program is further described in detail, using a comprehensive overview of the TIDieR (5) which includes 7 main topics: ‘Why’, ‘What’, ‘Who provided’, ‘How’, ‘Where’, ‘When and How much’ and ‘Tailoring’. Particularly the first topic ‘Why’ is discussed in detail, as it provides the rationale for the program.

### 3.1 Why

The development of the AHA-BOOST program was based upon the principles of the Bobath concept (8) and this program and concept were used in the AHA-BOOST pilot study (9). To structure our clinical reasoning process, the Model of Bobath Clinical Practice (MBCP) is used (6). The MBCP is a structured tool designed to support clinical reasoning in neurorehabilitation. It was developed through expert consensus using focus groups of experienced neurorehabilitation clinicians and is grounded in the Bobath concept—a widely applied clinical framework in neurorehabilitation (6). The MBCP provides a standardized approach for guiding therapeutic decisions in patients with acquired brain injury, including stroke. This clinical reasoning process is patient centered, meaning that goals and therapy are set in communication with the patient. A working hypothesis is developed based on a task-related functional movement analysis, which is conducted by a multidisciplinary team using observational assessment, clinical outcome measures, and reflection on the patient’s reaction to manual, verbal, and environmental facilitation. This hypothesis considers the movement diagnosis (‘how is the patient deviating from efficient/typical movement?’) and identifies the potential of the patient and as such forms the basis for therapy. Assessing typical and efficient movement post stroke includes evaluating motor control, biomechanical patterns, movement performance, symmetry, etc. In addition to observational assessment, clinical outcome measures are used (10). The outcome of the treatment (dis-) confirms the hypothesis and continues the iterative process of clinical reasoning. The MBCP was selected for several reasons. First, it offers a structured framework for clinical reasoning to plan therapy based on a patient’s functional potential. Second, it promotes a holistic, patient-centered approach that considers multiple domains of functioning (motor, sensory, cognitive) and supports interdisciplinary collaboration. Lastly, the Bobath concept, on which the MBCP is based, is commonly used in clinical settings. Although its superiority over other rehabilitation models remains debated, it continues to be widely accepted and clinically relevant in stroke rehabilitation (6). And while it is not a classical outcome measure and has not yet undergone formal psychometric validation, its clinical relevance and structured use in stroke rehabilitation are well-known (11).

Skilled grasp is a sensorimotor process, requiring the brain to extract cues from the environment to shape a motor command (12). Therefore, reaching and grasping an object requires the processing of its precise location with respect to the hand, the integration of the object’s intrinsic properties (such as size and shape of the object) and the integration of the goal of reaching and grasping. This information needs to be transformed into guiding the hand towards the object and shaping the hand for efficient grasp of the object in relation to the required goal, whilst considering the biomechanical interactions all along the multi-articulate anatomical chain, linking the proximal arm to wrist, hand, and fingers (12).

**Figure 1:**
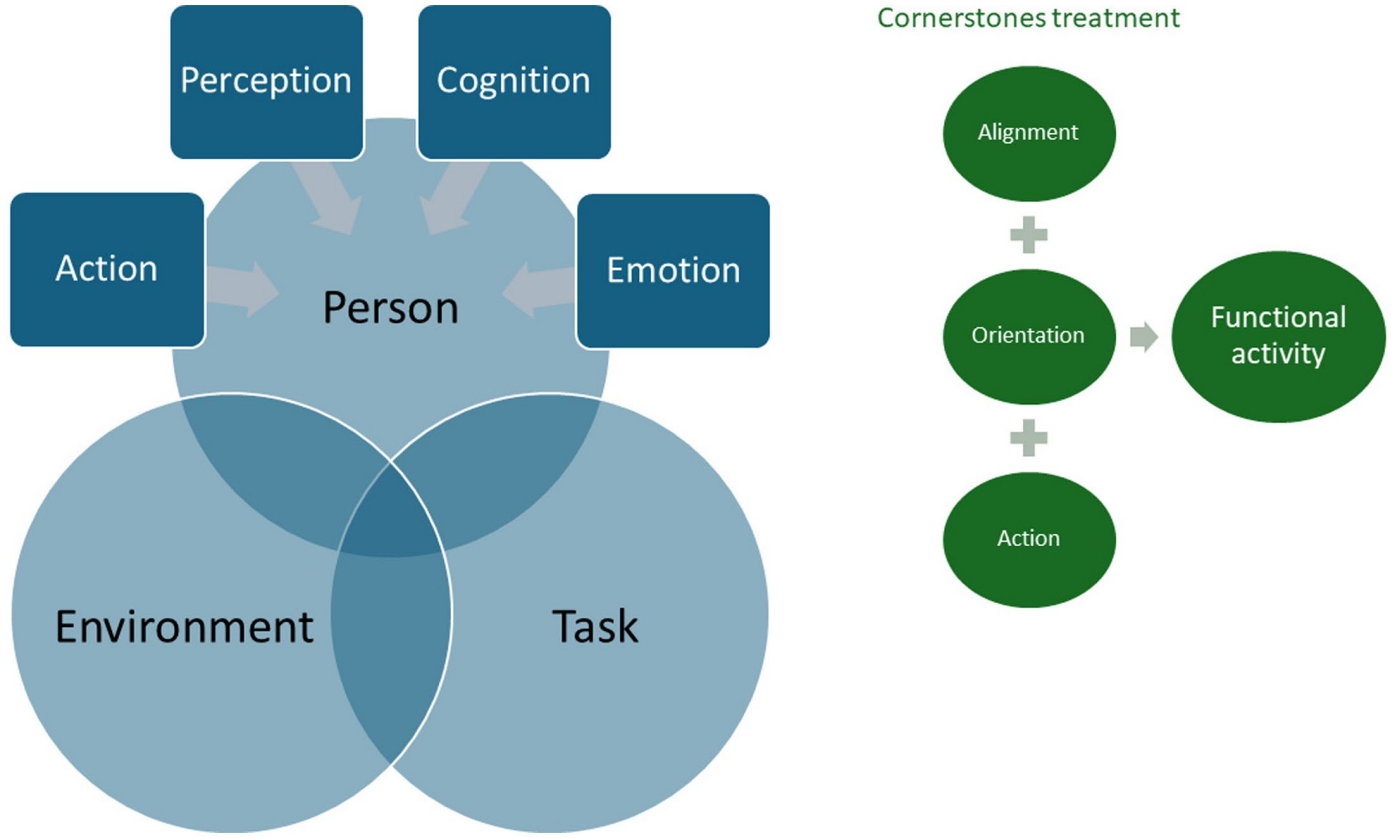
Building blocks of efficient movement, according to Shumway-Cook and Woollacot (13).

As presented in Figure 1, in daily life a person is performing a task in a chosen environment. The motor activity of the person is defined by the characteristics of the environment, the characteristics and the goal of the task and the ability of the person. When looking at the skills and abilities of the person, aspects of action, perception, cognition, and emotion must be considered (13). All these aspects need to be assessed and addressed to influence the efficiency of the task: (1) *Action*: Am I able to recruit muscles/synergies in an efficient way (intensity and selectivity)? How about the role of movement planning and the activity of the medial and lateral descending system?; (2) *Perception*: Am I able to inform my central nervous system by using my senses and receptors (skin receptors, proprioceptors, graviceptors, vestibular system, vision, hearing, and their connection with relevant areas in the brain, such as thalamus, parietal cortex)?; (3) *Cognition*: Am I able to understand the task, how it is performed, do I recognize the environment, am I able to be attentive, can I execute a task? In other words, how am I coping with neuropsychological disorders connected to the associative area’s in the brain?; and (4) *Emotion*: Do I recognize aspects of fear, nervosity, (lack of) drive (13)?

Finally, to reach efficiency in a functional activity during treatment, the person needs to be properly aligned, oriented and activated: (i) *Alignment*: is related to a proper biomechanical relation of bones, joints and muscle length in relation to the upcoming task; (ii) *Orientation*: is related to informing the brain about the body and its relationship to the environment (How is my body related to the base of support and related to the upcoming task, where is the object in relation to my hand, where is my arm/hand, what is the aim of the task, …). This gives us information about the integrity of the sensory system (receptors, ascending pathways and processing in, among others, the thalamus, parietal cortex and cerebellum); and (iii) *Action*: Is related to recruiting the relevant muscles in an ordered way (such as posture before movement and activating synergies). Here we generate information about the integrity of, for example, the frontal cortex, the cerebellum and the medial and lateral descending tracts.

People with stroke have impaired postural control; for example, in sitting, these individuals have more displacements of the center of pressure in both the anterior–posterior and medial-lateral directions (14). People with stroke are lacking orientation due to an impaired body scheme or malorientation in relation to the base of support. Additionally, most patients experience difficulties in activating muscles due to paresis. Considering only disability derived from upper-limb (UL) impairment, more than 75% of people with stroke remain with upper-limb impairment at the chronic stage, which renders the rehabilitation of the ULs after stroke a challenge (15). Over fifty non-conventional interventions aimed to improve upper limb recovery after stroke were evaluated by Saikaley et al. (16). Among these, modified constraint-induced movement therapy showed the highest probability of being the most effective (16). However, The Cochrane review by Pollock et al. (17) reported only moderate-quality evidence for constraint-induced movement therapy (CIMT) on ADL outcomes and concluded that no intervention currently used as routine practice is supported by high-quality evidence (17). Additionally, implementing CIMT in clinical practice shows several barriers: it seems only suitable for a very specific subgroup of stroke survivors and the therapy requires intensive therapeutic resources (18). To address this treatment gap and provide effective intervention for a broader population of stroke survivors, the arm-hand BOOST program (AHA-BOOST) was developed.

The JSU diagram was developed based on the hypothesis that not every stroke patient needs the same approach in treating the upper limb (4). Stratifying patients based on their level of impairment is the prerequisite for providing patient-tailored treatment for the upper limb. The AHA-BOOST program focuses on the mild to moderately impaired patient after stroke for which cut-off scores in 2 items of the FMA-UE (19) are used. Stage two is referring to the synergistic movement of the arm whilst stage five refers to hand function. Patients are included in the AHA-BOOST program if a score of 8–17 is achieved on stage 2 (volitional movement within synergies) of part A, or a score of <8 on stage 2, part A, in combination with a score >6 on stage 5 (hand) of part C. This means that patients can join the program if they can use their upper limb in a flexor or extensor synergy, or when they show some hand function (4, 19).

The key aspects of the AHA-BOOST program include the integration of knowledge on:

1. Neurophysiology: understand the system—search for the potential
2. Sequences of reaching and grasping: from abdominal core to hand—and back
3. The brain perceives the arm as heavy—and acts as such
4. Orientation of the hand towards the object defines the movement of the arm

In the following sections, we will explain these four key aspects, provide the theoretical background and translate each of them into clinical practice.

#### 3.1.1 Neurophysiology: understand the system—search for the potential

The primary problem after stroke is not in the body, but in the brain. Therefore, to treat patients effectively, it is necessary to understand as much as possible how the brain works and how we can ‘speak to’ and ‘change’ the brain to have a better motor outcome. The better we understand the brain and its deficits, the better we will be able to search for the (residual) potential.

Neurophysiology-based therapy starts by identifying the potential of the neurological system. Within this concept, four underlying elements are discussed: (1) postural control, (2) postural adjustments, (3) functional synergies, and (4) the neural grasping circuit.

##### 3.1.1.1 Postural control

Postural control is a prerequisite for movement initiation and can be achieved through strategies such as adopting an active starting position or modifying the environment. In neurophysiology, this concept is reflected in the Henneman’s size principle (20), which describes the recruitment of motoneurons in the spinal cord: motoneurons with small cell bodies (tonic, slow, fatigue resistant) are recruited before neurons with larger cell bodies (phasic, fast, and fast fatigued). This order is also followed for efficient movement so here stability (small) precedes mobility (large) (21). In individuals post-stroke, disrupted descending control may alter recruitment order and result in inefficient, compensatory movements (22, 23). Therefore, during therapy, exercises are selected to activate different stabilizing muscles through small and controlled movements. Gradually, larger movements are incorporated as patients become capable of maintaining stability for longer periods.

Postural control is the coordinated, sequenced organization of stability and mobility of the multi-link kinetic chain to maintain, achieve or restore balance for efficient performance of a motor task. Postural control is a complex skill which controls the body’s position for purposes of orientation and stability (24). The quality of movement performance is considered with respect to the integration of postural control and selective movement, the active alignment of all body segments and the ability to receive, integrate and respond to sensory information (8).

**Figure 2:**
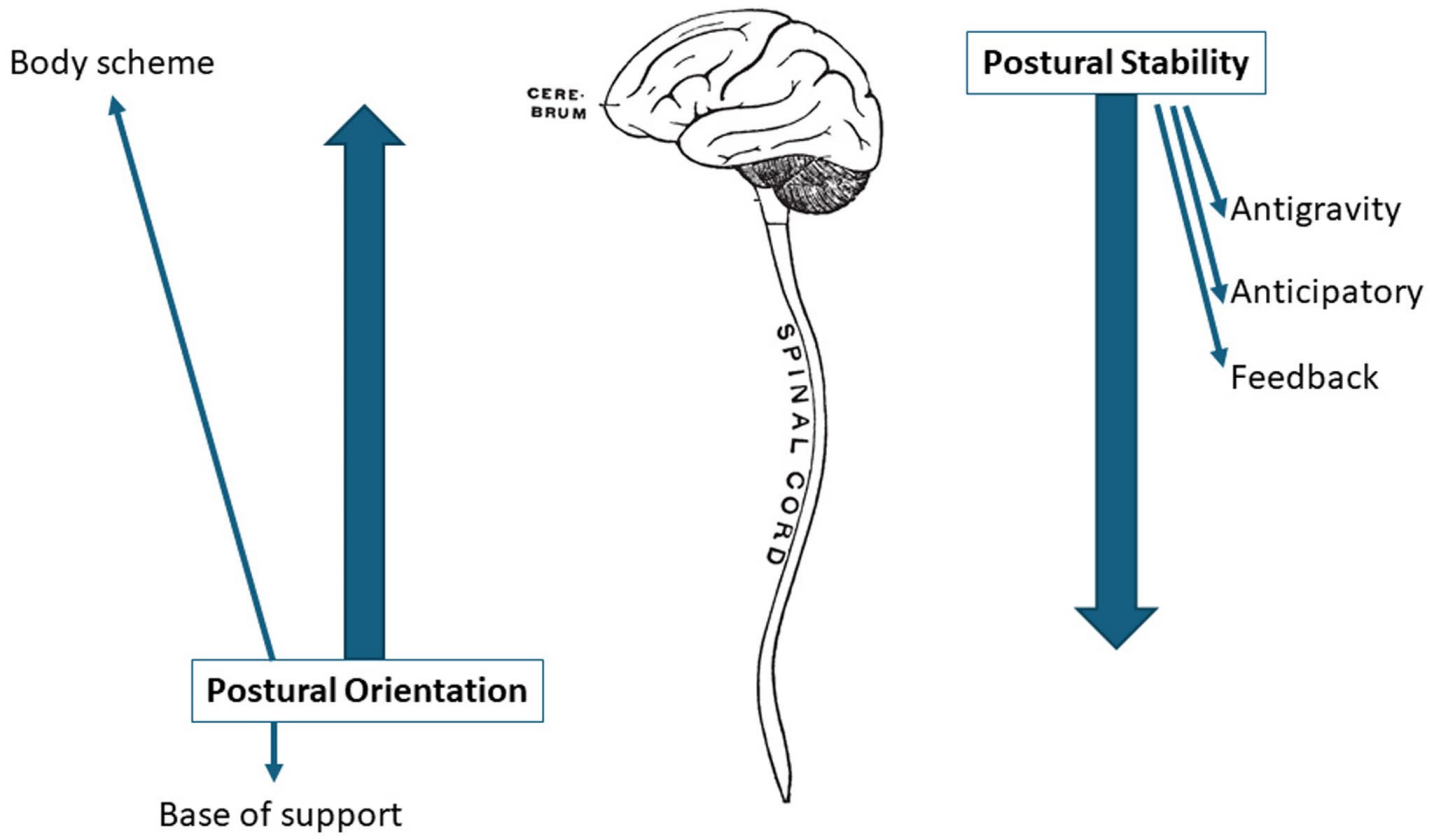
Integrated postural control mechanism.

Figure 2 shows the integrated postural control mechanism. Postural orientation involves the active control of body alignment and tone with respect to gravity, support surface, visual environment, and internal references (body scheme). It is influenced by receptive information (including vision, somatosensation and vestibular system) to inform the relationship with the base of support and the body scheme. Postural stability is related to coordinating movement strategies (antigravity activity, anticipatory and feedback mechanisms) to stabilize the center of mass during self-initiated and externally triggered disturbances (25). The specific response strategy selected depends on the characteristics of the external postural displacement but also on the individual’s expectations, goals, and prior experiences.

##### 3.1.1.2 Postural adjustments related to reaching and grasping

**Figure 3:**
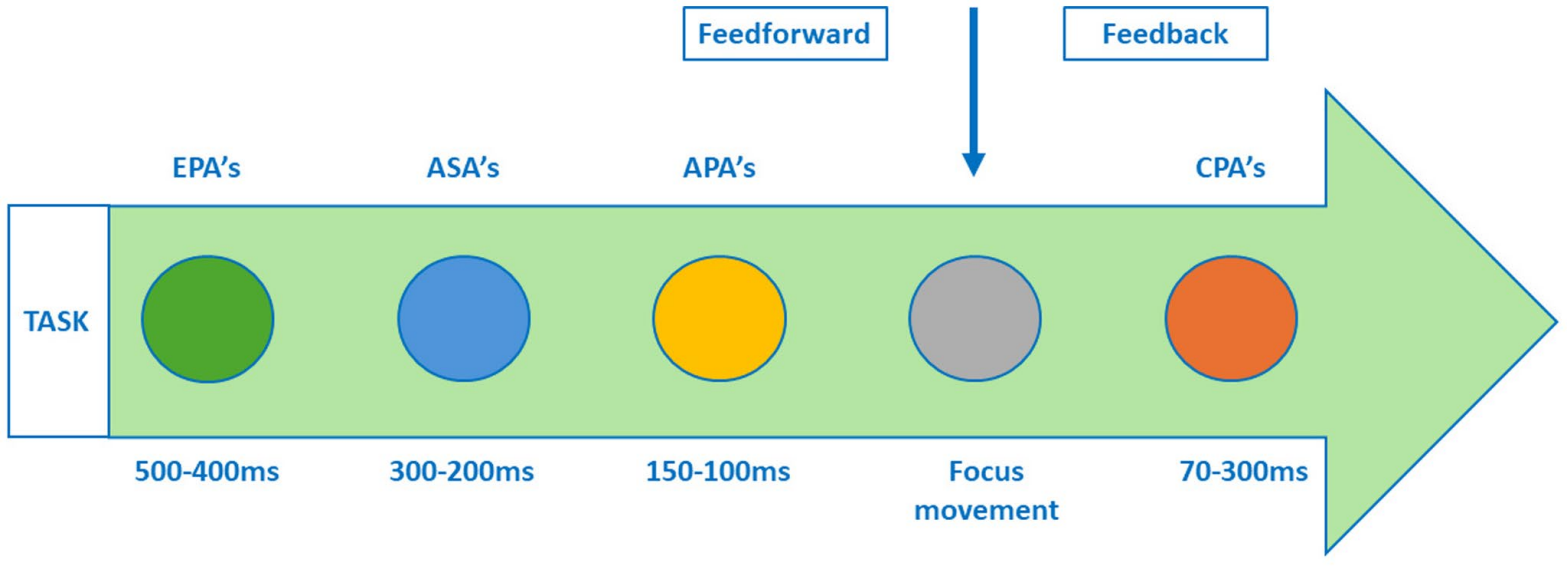
Postural adjustments connected to voluntary movement, according to Caronni and Cavallari (27), Krishnan et al. (29) Latash and Huang, (30), Piscitelli et al.(31), and Aruin (32). EPA’s, Early Postural Adjustments; ASA’s, Anticipatory Synergistic Adjustments; APA’s, Anticipatory Postural Adjustments; CPA’s, Compensatory Postural Adjustments.

According to the systems approach theory of motor control, movement arises from the interaction of multiple body systems, the task, and the environment, rather than being controlled by a single central command or specific muscle group (26). Within this framework, different postural adjustments are required, which occur around voluntary movements. More specifically, anticipatory postural adjustments (APAs) ensure balance and create a chain of stability for prime movers (27). The package of anticipatory adjustments creates the necessary conditions for efficient center of mass displacement within the base of support in each condition (28). The different postural adjustments related to reaching and grasping are shown in Figure 3.

Different postural adjustment are identified, which occur in the following order around the voluntary movement: (1) Early postural adjustments (EPA): Adjust the posture in preparation to the expected perturbation. It occurs prior to anticipatory postural adjustments (APA) (29); (2) Anticipatory synergistic adjustments (ASA): Reduce stability in preparation to quick actions (30, 31); (3) Anticipatory postural adjustments (APA): are unconscious muscular activities preceding the voluntary movement aiming to compensate the changes in posture produced by the focal movement itself (27); and (4) Compensatory postural adjustment (CPA): Initiated by the sensory feedback signals and serve as a mechanism of restoration of the position of the center of mass after a perturbation has already occurred (32). They restore balance following an unexpected perturbation.

The APA’s are important in reaching and grasping. As mentioned in (33), clinically, they make the upper limb feel ‘light’ and ‘effortless to move’ when the individual reaches. The proximal trunk provides stability, which is the foundation for the shoulder muscles to then take the hand forward efficiently (33). As such, building a postural background for reaching is the prerequisite of facilitating the efficient sequence of movement to fractionate reaching and achieve adequate hand positioning (Box 1).

BOX 1 **Clinical relevance of postural control and postural adjustments related to reaching and grasping in AHA-BOOST program**

Balance and postural disorders are among the most prevalent consequences after stroke (34) and limit the execution of several daily activities (35). The integration of posture and movement utilizes anticipatory and reactive postural control mechanisms, both of which are modulated by sensory inputs and influenced by learning and experience (36). Appropriate postural control and the ability to move selectively facilitate the production of coordinated sequences of reaching and grasping. Postural control and selective movement patterns are interdependent and enable movement efficiency. Restoration of postural control by influencing postural orientation (providing structured sensory and proprioceptive information) and postural stability (building antigravity activity to enable postural feedforward and feedback processes) allows more selective and efficient use of the upper limb (37, 38).

##### 3.1.1.3 Functional synergies

Taks-specific muscle synergies are neurophysiological entities whose combination, orchestrated by the motor cortical areas and the afferent systems, facilitates motor control and motor learning (39). A limited set of available muscle synergies may handle effectively musculoskeletal redundancy and simplify motor control (40). Synergies are built out of spinal units, which are functional units of spinal interneurons which generate specific motor outputs by imposing a specific pattern of muscle activation (39, 41). Different cortical and subcortical tracts influence the propriospinal network, which is responsible for the organization of normal upper limb synergies. Pathological synergies of the upper limb will occur when the cortical influence on the propriospinal network alters due to a stroke. By treating the upper limb, it is necessary to explore the potential of the brain to restore the cortical influence on the brainstem nuclei such as the reticular formation, to turn the pathological synergies into more efficient, normal movements. Retraining non-pathological movement patterns can simplify motor control and select the appropriate muscles for a behavioral goal (39, 40). A more in-depth understanding of this organization can be found in Alstermark and Isa (41).

After a stroke, altered descending commands from the cortical and subcortical areas to the brain stem and spinal cord generate abnormal motor behavior through faulty activation of the spinal modules (39). Individuals with moderate to severe post-stroke hemiparesis cannot control proximal and distal joints of the arm independently because they are constrained to stereotypical movement patterns called flexion and extension synergies. Accumulating evidence indicates that these synergies emerge because of upregulation of diffusely projecting brainstem motor pathways following stroke-induced damage to corticofugal pathways (42). The neurological deficits after stroke are not simply weakness or paralysis but also improper coordination between limb segments, which partly is due to improper functioning of the motor cortical inhibitory network (43). The integrated nature of motor cortical control strongly suggests that neurorehabilitation programs should be aimed at reinforcing complete tasks, such as reaching to grasp and working with complete movement synergies (43) (Box 2).

BOX 2 **Clinical relevance of synergies in AHA-BOOST program**

The goal of therapy is to regain task-related reach and grasp. Assessment and treatment of reaching and grasping should be aimed at inhibiting pathological synergies and to facilitate more efficient synergies. This can be achieved by treating postural control (see before) to relate stability to mobility, by respecting proximal stability as part of the sequences of reaching and grasping (see below) and by de-weighting the upper limb (see below). Congruent with motor skill learning principles (44), synergies can be influenced by adapting the difficulty of the task or by modifying the environment so that it determines the trajectory of the hand and arm.

##### 3.1.1.4 The neural grasping circuit

**Figure 4:**
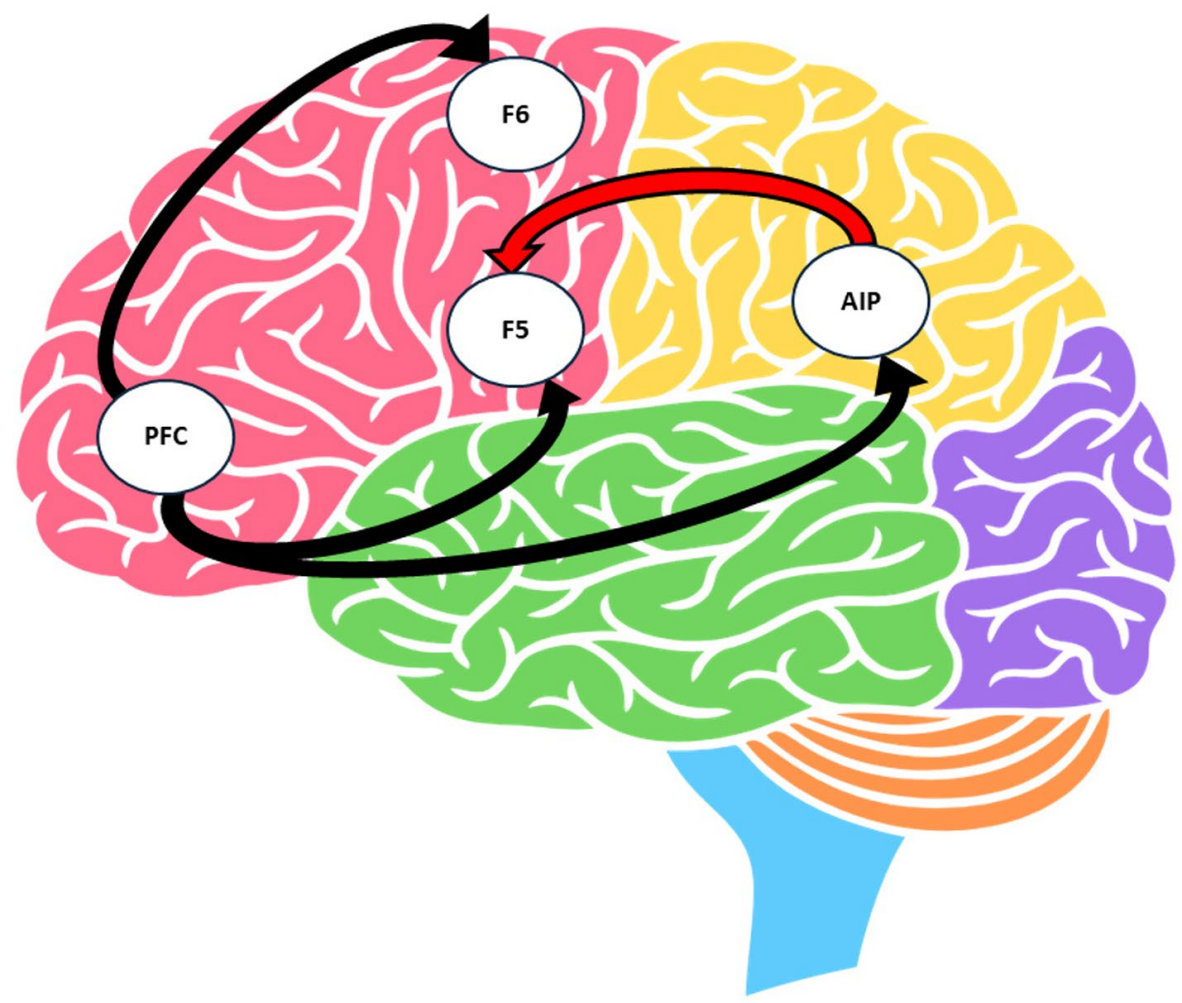
The neural grasping network, according to Gerbella et al. (46). F5, ventral premotor area; F6, pre-supplementary area; PFC, prefrontal cortex; AIP, anterior intraparietal area.

Figure 4 illustrates the neural grasping circuit, which supports the transformation of visual object information into goal-directed hand movements. Grasping an object requires precise control of hand and finger movements, including the ability to shape the hand precisely before contact. Lesions of the precentral motor cortex can lead to flaccidity, force deficits, and, most importantly, severe impairments in individual finger movements (45). The core sensorimotor transformation circuit involves the anterior intraparietal area (AIP) and the ventral premotor area F5 and plays a primary role in visuomotor transformation for the selection and control of the appropriate grasping motor acts (46). The AIP is responsible for processing object-related visual features and transmitting this information to area F5, which contains a storage of preformed motor actions. These stored motor actions include general goals (e.g., grasping or holding), specific grip patterns (e.g., configuration of fingers necessary for precision), and temporal components (e.g., hand opening). This organizational structure in F5 facilitates rapid and accurate matching between the object’s visual properties and the required grasping action. For example, the size of a cup is recognized by AIP, which connects to F5 that contains a storage of motor actions connected to precise shaping of the hand before touching the object (45). Once the appropriate grasping movement is selected, there are other factors that may modulate hand shaping and grip force during grasping. These are the visually detectable physical properties of the object such as texture or slipperiness; and other properties related to previous experience with the same object (46). Therefore, by incorporating everyday, known objects, it can be assumed that the neural grasping circuit will be activated and might facilitate the use of functional movement patterns. A recent study by Lacerernza et al. used functional near-infrared spectroscopy (fNRIS) to measure these functional activations of the motor cortex during arm-raising actions. The findings of this study may provide a better understanding of the neural mechanisms underlying goal-oriented tasks in humans (47) (Box 3).

BOX 3 **Clinical relevance of the neural grasping network in AHA-BOOST program**

Involve the patient in defining relevant tasks and create a motivating and interesting environment. Practice in the most realistic setting possible with everyday objects. This approach is in line with motor learning principles (44) and knowledge about using enriched environment in motor learning (48).

#### 3.1.2 Sequences of reaching and grasping: from abdominal core to hand—and back

Reaching and grasping can be described in the following sequences: Abdominal core—Scapula setting—External rotation of glenohumeral joint—Synergistic reaching activity with triceps activation and biceps reciprocal inhibition to move the hand away from the body—Orientation and activation of the hand, with the hand being the reason to move the arm. Here too, the connection between stability and selectivity plays an important role. That is why we distinguish three cores (abdominal core, scapula setting and core of the hand) as a background for selective movement of the arm and hand. Creating this background increases the probability to reach the best possible potential in sequences of reaching and grasping. It should be considered that there are infinite possibilities for movements as possible movement patterns.

Controlling the abdominal core gives a stable background for *M. serratus anterior* as prime mover for scapula setting. Scapula setting brings the external rotators of the glenohumeral joint in a biomechanical ideal position to work. As such, the triceps is positioned in a good alignment to be active and extend the arm to then transport the hand towards the object. The core of the hand is built by the stability of the wrist and the activity of the intrinsic hand musculature. It is the prerequisite for selective finger movement. Proprioceptive representations of the hand encode time-varying joint postures distributed over the entire hand. This neural representation stands in contrast with its proximal limb counterpart, which preferentially encodes movement (49). Given the fact that the core of the hand is mostly build by the intrinsic musculature of the hand, mobilization and activation to preserve the arches of the hand is of utmost importance. In this way, the hand can take its postural role as background for selective finger movements.

The key elements of the reaching and grasping sequence are: (i) the abdominal core; (ii) scapula setting and external rotation of the glenohumeral joint; and (iii) *M. triceps brachii* activation to ensure a long arm (Box 4).

##### 3.1.2.1 Abdominal core

In healthy populations the individual elements of the ‘stabilization synergy’ (*M. transversus abdominus*, diaphragm, pelvic floor, deep fibers of lumbar *M. multifidus*) co-activate in advance of limb movement (50). A functional definition of core control is “the ability to generate optimal intra-abdominal pressure to support both breathing and the provision of three dimensional postural and movement control of the torso and particularly control of the pelvis on the legs and scapula on the arms” (50). The stabilization synergy or core control is about coordination rather than about strength and needs coordinated and co-activated work of the ‘intrinsic core’ (*M. transversus abdominus*, diaphragm, pelvic floor, deep fibers of lumbar *M. multifidus*), on which a coordinated work between the ‘intrinsic’ and ‘extrinsic’ core (*mm Erector spinae*, *m rectus* *abdominis*, *mm obliquus externus and internus*, *m Iliopsoas*) is build (51). Real ‘core control’ comes from uptraining the intrinsic core muscles. Activating the abdominal muscles helps stabilizing the trunk, providing a proximal base for distal mobility during reaching (52).

The neurophysiology related to abdominal core synergy involves how the central nervous system (CNS) organizes feedforward and feedback mechanisms to stabilize the trunk during movement. This process includes sensorimotor integration, motor planning, and specific muscle recruitment patterns (53).

In people with stroke the abdominal core can be activated in an inefficient way due to a change of functioning of utmost the medial (pontine) and lateral (medullary) reticulospinal tract, which connect to the axial (and proximal) stabilizing musculature (54–56).

##### 3.1.2.2 Scapula setting and external rotation of the glenohumeral joint

Optimal function of the shoulder complex is created by the sequential action between proximal stabilizers and distal movers.

A proper scapula setting needs activation of *M. serratus anterior* (1), lower and upper *M. trapezius* (2) and middle *M. trapezius* (3). These are called the proximal stabilizers (57). These muscles orientate the cavitas glenoideale to cranial, lateral, and anterior which is positioning the glenohumeral joint in a biomechanical ideal position to activate the external rotators of the shoulder. As such, a stable background is created to activate the distal movers: *M. deltoideus*, M. supraspinatus, *M. biceps brachii* and *M. tricpes brachi*.

In people with stroke, recruitment patterns, measured by EMG, are characterized by delayed activation and early inactivity of *M. serratus anterior*, which leads to improper scapula setting and inefficient scapular dynamics (58). Therefore, the rotator cuff loses its capacity to stabilize the glenohumeral joint and utmost external rotation of the glenohumeral joint is prohibited. The difference in muscle activation may be the reason for the stereotype joint movement of upper limb flexion synergy. Spinal interneurons might be responsible for this phenomenon. It was found that motor impulse after stroke could only activate interneurons through other descending pathways, such as the reticular spinal tract and simultaneously activate all motor neurons connected to the interneuron (59).

##### 3.1.2.3 *M. Triceps brachii* activation to ensure a long arm

The *M. triceps brachii* has an agonist activity in horizontal reaching (60). The intensity of this activity becomes stronger when the target is further away from the body. If the movement of the arm is increasing towards 45° anteflexion, the role of the posterior *M. deltoideus* becomes more apparent. EMG activity during horizontal reaching has shown the agonist action of the *M. triceps brachii*, followed by antagonist activity of *M. biceps brachii* and *M. brachioradialis*. Before this latter, the posterior part of the *M. deltoideus* assists in bringing the triceps in a proper position (60).

BOX 4 **Clinical relevance of the sequences of reaching and grasping in AHA-BOOST program**

Assessment and treatment of the ‘three cores’ (abdominal core—scapula setting—core of the hand) is the basis to create sequenced movement. Whilst assessing the patient, any of these sequences can be efficient or inefficient. Therefore, the decision where to start the treatment is based on the potential of the patient. In some patients, scapula setting can be created by using the potential of the hand to activate *M. triceps brachii* and in doing so to influence scapula setting. The access to sequences of reaching in other patients might be the potential of scapula setting which facilitates activity in the *M. triceps brachii* and offers access to activating the hand. Sequences of reaching is strongly connected to creating postural control, synergies, de-weighting the arm and the leading factor of the hand.

#### 3.1.3 The brain perceives the arm as heavy—and acts as such

In people with stroke, the flexor synergy can be seen as a normal adaptation to an abnormal situation. Lifting the arm to reach, can be compared to healthy subjects when they lift a heavy weight. To lift this weight, healthy people are using a kind of flexor synergy pattern. This observation led to the hypothesis that the arm in people with stroke is perceived as heavy and therefore patients move their arm in a flexion synergy pattern when reaching.

Weight support facilitates greater training dosages and can also improve movement quality (61) during reaching tasks. It was shown to have the largest effect on the quality of reaching and grasping in the moderate to severe motor impairment group (FMA-UE < 50) (61). Weight Support reduces antagonist muscle activity in both healthy elderly and chronic people with stroke (62). Abnormal coupling of joint torques between the shoulder and elbow is also decreased by using weight support (22, 63). Increased shoulder abduction recruitment increases contra-lesional motor cortical activity and the recruitment of anatomically diffuse reticulospinal motor pathways, which is not advantageous (23). The stereotyped flexor synergy can thus be mitigated with weight support, permitting greater elbow extension and access to the reaching workspace (64–66). One should take into consideration that these results are based on studies with small sample sizes (Box 5).

BOX 5 **Clinical relevance of weight support in AHA-BOOST program**

De-weighting of the arm is initiated by proximal stability (abdominal core and scapula setting) and can be further achieved by adding environmental adaptations to support the arm and by specific types of bandaging the arm and hand towards supination and neutral wrist position. Then, initiating reaching by facilitation through the bandage can achieve adequate reach execution.

#### 3.1.4 Orientation of the hand towards the object defines the movement of the arm

Movement planning and execution depends on the orientation of the hand and how it will interact with the environment to complete a specific task. The hand leads the reaching movement. The scapula is subservient to the hand in most tasks. It has been shown that scapula setting occurs by correct orientation of the hand. Therefore, to influence proper stability and movement of the scapula, the hand should always be involved (67).

In people with stroke pronation, palmar wrist flexion and flexed fingers are common. This position orientates the elbow towards flexion and the shoulder toward internal rotation and abduction. Re-orientation towards a more stable position of neutral between pro-and supination, a stabilized wrist and active core of the hand, will influence better orientation of the shoulder (towards more external rotation and less abduction) as a prerequisite to influence efficient sequence activation (Box 6).

BOX 6 **Clinical relevance of orientation of the hand in AHA-BOOST program**

Correct orientation of the hand facilitates the sequences of reaching. Through creating the core of the hand, neutral wrist position and supination, more efficient reaching synergies are facilitated. These synergies can be reinforced by increasing intensity and repetition.

### 3.2 What

See materials and equipment.

### 3.3 Who provided

The AHA-BOOST group sessions are conducted by a team of specifically trained therapists, including both occupational therapists and physiotherapists. Prior to delivering the intervention, they undergo extensive training in AHA-BOOST therapy.

The training covers the following topics:

- Theoretical Component: In-depth exploration of the theoretical background and key principles underlying this new approach.
- Practical Workshops: Hands-on workshops designed to facilitate learning and application of the key principles with various patients.
- On-site Support: Valuable insights and support provided at the rehabilitation centre to optimize therapy application for different patients.

### 3.4 How

The program consists of twenty, one-hour group sessions (spread over 4 weeks, 5 days a week) and 1 h of individual robot-assisted therapy per week. The group sessions are executed with a maximum of four participants, under supervision of two experienced therapists (both an occupational and physiotherapist).

### 3.5 Where

The program is provided in an inpatient rehabilitation setting. No specific infrastructure is needed.

### 3.6 When and how much

The AHA-BOOST intervention is developed for patients in the sub-acute phase post stroke and the program consists of a total of 24 h of additional therapy delivered over a period of 4 weeks. These 24 h are divided in twenty, one-hour group sessions and 4 h of robot-assisted hand therapy. This means that patients will receive every day 1 h of BOOST therapy and weekly 1 h of robot-assisted therapy. The dose was determined based on clinical feasibility (as it concerns additional therapy, on top of the usual care therapy dose) and current practice standards in an inpatient rehabilitation setting, while still offering sufficient intensity to potentially elicit functional improvement. This is further supported by the recent European Stroke Organisation (ESO) guideline on motor rehabilitation with a recommendation to provide an additional minimal dose of 20 h of repetitive upper limb practice to improve arm capacity (68).

### 3.7 Tailoring

AHA-BOOST is a patient-centered approach based on the individual abilities and treatment goals of the patient. Exercises are structured around the four key aspects of AHA-BOOST, with difficulty levels gradually increasing. Each of the interventions is tailored to the individual patient, based upon the ongoing assessment of the therapists by means of the Model of Bobath Clinical Practice (MBCP) (6), discussion within the group of therapists and individual treatment goals of the patient.

## 4 Results and discussion

In 2019, a phase II RCT was conducted at Jessa Hospital (9) to assess safety, feasibility and first signs of effectiveness in which the AHA-BOOST program was compared to a dose-matched control program of lower limbs and general reconditioning exercises (leg press, quadriceps bench, sitting bike, exercise program for lower extremities). Both interventions were provided in addition to conventional inpatient rehabilitation. The results of the pilot RCT showed that the AHA-BOOST program, on top of usual care, is feasible and safe in the sub-acute phase post stroke and suggests positive, clinical meaningful effects on upper limb function (80% of patients of the experimental group reached the minimal clinical important difference on ARAT and FMA-UE), especially when delivered in the early sub-acute phase post stroke.

Recently, a phase III RCT started including a clinical, health economic and process evaluation of the AHA-BOOST program. Recruitment of participants started in June 2024 (target *n* = 80) from different inpatient stroke rehabilitation centers in Belgium: rehabilitation center K7 of university hospital Gent, general hospital Maria Middelares, rehabilitation center Revales, rehabilitation campus Pellenberg of university hospital Leuven, and rehabilitation center RevArte, and follow-up measurements are planned until April 2027. A multicomponent utilization strategy is included in the project focusing on (1) a post-graduate training package educating AHA-BOOST to interdisciplinary teams; (2) preparing an argumentation portfolio for implementation of AHA-BOOST in stroke care; and (3) a blueprint for implementation of AHA-BOOST by rehabilitation center management.

## 5 Summary

This overview provided a detailed description of the AHA-BOOST therapy program, which was developed at Jessa Hospital, rehabilitation center in Herk-de-Stad, Belgium. We focused on the theoretical background and development of the program, using a comprehensive overview of TIDieR (5). The AHA-BOOST therapy program is developed for patients with mild to moderate impairments in the upper limb after stroke. It offers a four-week tailored treatment program consisting of daily one-hour group sessions and weekly individual robot-assisted therapy sessions. The individual robot-assisted therapy is provided to increase dosage and intensity of the intervention, which can further boost recovery. The group sessions are built around four key aspects of AHA-BOOST, namely neurophysiology, sequences of reaching and grasping, de-weighting of the arm and orientation of the hand towards objects.

In a phase II RCT (9) the AHA-BOOST program proved to be feasible and safe and suggests positive, clinical meaningful effects on upper limb function. A phase III RCT including a clinical, health economic and process evaluation of the AHA-BOOST program is currently ongoing.

## Data Availability

The original contributions presented in the study are included in the article/supplementary material, further inquiries can be directed to the corresponding author.
